# Enhanced Routing Algorithm Based on Reinforcement Machine Learning—A Case of VoIP Service

**DOI:** 10.3390/s21020504

**Published:** 2021-01-12

**Authors:** Davi Ribeiro Militani, Hermes Pimenta de Moraes, Renata Lopes Rosa, Lunchakorn Wuttisittikulkij, Miguel Arjona Ramírez, Demóstenes Zegarra Rodríguez

**Affiliations:** 1 Department of Computer Science, Federal University of Lavras, Minas Gerais 37200-000, Brazil; davi.militani@estudante.ufla.br (D.R.M.); hermes@ufla.br (H.P.d.M.); renata.rosa@ufla.br (R.L.R.); 2 Wireless Communication Ecosystem Research Unit, Department of Electrical Engineering, Chulalongkorn University, Bangkok 10330, Thailand; lunchakorn.w@chula.ac.th; 3 Department of Electronic Systems Engineering, University of São Paulo, São Paulo 05508-010, Brazil; maramire@usp.br

**Keywords:** routing algorithms, machine learning, reinforcement learning, intelligent routing, VoIP, QoE

## Abstract

The routing algorithm is one of the main factors that directly impact on network performance. However, conventional routing algorithms do not consider the network data history, for instances, overloaded paths or equipment faults. It is expected that routing algorithms based on machine learning present advantages using that network data. Nevertheless, in a routing algorithm based on reinforcement learning (RL) technique, additional control message headers could be required. In this context, this research presents an enhanced routing protocol based on RL, named e-RLRP, in which the overhead is reduced. Specifically, a dynamic adjustment in the Hello message interval is implemented to compensate the overhead generated by the use of RL. Different network scenarios with variable number of nodes, routes, traffic flows and degree of mobility are implemented, in which network parameters, such as packet loss, delay, throughput and overhead are obtained. Additionally, a Voice-over-IP (VoIP) communication scenario is implemented, in which the E-model algorithm is used to predict the communication quality. For performance comparison, the OLSR, BATMAN and RLRP protocols are used. Experimental results show that the e-RLRP reduces network overhead compared to RLRP, and overcomes in most cases all of these protocols, considering both network parameters and VoIP quality.

## 1. Introduction

Nowadays, there is a great demand for internet application services, such as video [1] and audio streaming, Voice-over-IP (VoIP) [2,3], online games [4] among others. Multimedia services represent more than 50% of current Internet traffic [5]. VoIP service is one of the most popular communication services due to the low phone call rate compared to conventional telephony [6], but also due to the high speech quality level achieved in recent years [7]. Thus, network providers need to perform monitoring and operation tasks to ensure an acceptable end-user’s Quality-of-Experience (QoE).

In ad-hoc wireless networks, to ensure a reliable network performance is a great challenge due to the characteristic of this kind of network [8]. Dynamic topology, shared wireless channels, and limited node capabilities are factors that need to be considered in order to provide a high quality VoIP service. For instance, device batteries are limited resources that can lead to link losses connected to that nodes during power failures [9].

In a VoIP communication, end-user’s QoE is determined by the user’s perception [10,11,12]. In general, speech quality assessment methods can be divided in subjective and objective methods. Subjective methods are performed in a laboratory environment using a standardized test procedure [13]. Several listeners score an audio sample and the average value is computed and named Mean Opinion Score (MOS). However, subjective methods are time-consuming and expensive [14]. Another manner to predict the quality of a VoIP call is through a parametric method, such as the E-model algorithms [15,16] which provides a conversation quality index estimated through different parameters related to acoustic environment, speech codec characteristics and network performance parameters.

Several factors, such as channel transmission capacity, node processing capacity, and routing protocols affect network performance parameters [17].

Conventional routing protocols in ad-hoc networks, such as Optimized Link State Routing (OLSR), are unable to learn from abnormal network events that occurred several times in the past [18]; then, those protocols can choose a path that in the past had recurrent problems. For example, let us consider a path *P* where a given node *N* presents recurrent shut downs due to either device failures or programmed power-offs to save energy [19]. If a conventional protocol chooses this path *P*, network degradation can occur, such as packet losses [20]. A routing protocol that is able to learn from previous network failure events could avoid this path improving the network performance. Hence, there is a need for protocols capable to learn from network data history. Therefore, it is important that routing protocols use strategies that make them learn from past experiences to choose optimal routing paths [21].

In the latest decades, Machine Learning algorithms have come to be used in several applications [22,23,24,25,26,27,28]. Thus, these algorithms can be applied into routing control protocols [29,30,31], specifically Reinforcement Learning (RL) is increasingly being used to solve routing problems [32,33,34]. In RL, an agent must be able to learn how to behave in a dynamic environment through iterations [35]. For instance, an agent who makes a choice receives a reward or a punishment whether the choice was good or bad, respectively. Hence, the RL technique can improve the steps along the decision making of path choice process, leading to better network performance, and consequently improved applications services, such as a VoIP communication [36].

In Reference [37], the authors introduce a generic model based on RL for ad-hoc networks focusing on routing strategies. Some works use RL for routing in urban vehicular ad-hoc networks (VANETs) [32]. Other works focus on wireless sensor networks and their characteristics [38] or unmanned robotic systems [39].

In Reference [18], an intelligent traffic control through deep learning is proposed, whose results demonstrated a performance gain compared to the traditional Open Shortest Path First (OSPF) routing protocol. In Reference [21], author uses Deep Reinforcement Learning to develop a new general purpose protocol, and obtained superior results compared to OSPF. However, both works do not focus on ad-hoc networks, and they do not compare the algorithm developed with ad-hoc network protocols. In Reference [40], a Reinforcement Learning Routing Protocol (RLRP) is proposed, which can be applied to ad-hoc networks.

Routing protocols require the use of control messages for their operation, they are responsible for the discovery of routes, for the dissemination of information on topology, among other things. However, control messages generate overhead on the network, thus decreasing network capacity especially in situations where the transmission channel may suffer interference or be saturated.

The use of RL technique in routing protocols may require an extra header, new control messages, or increasing the sending frequency of these messages. There are studies that aim to reduce overhead in traditional protocols. In protocols that use RL, a mechanism that provides the reduction of this overhead is relevant, because these routing techniques generate additional overhead.

In RL, there is an agent that interacts with an environment through the choice of actions [35]. In RL, each action generates a reward that generally defines whether the action taken was good or bad. In Reference [40], the rewards are sent to the nodes through control messages using a reward header that generates an overhead due to the use of RL. This additional overhead impacts on the global network performance.

In this context, there are research initiatives focused on decreasing the overhead originated by control messages. In Reference [41], authors propose an adjustment in the interval for sending hello messages of the AODV protocol in a Flying Ad-Hoc Networks (FANETs) scenario, focusing on reducing the energy consumption of unmanned aerial vehicles (UAVs) by reducing the frequency of sending the hello message.

The results show a reduction in energy consumption without loss of network performance. Despite presenting relevant results, the work focuses on FANETs and their specific characteristics. In Reference [42] the authors propose three algorithms to adjust the time to send Hello messages. The first algorithm is called Reactive Hello, where Hello messages are only sent when the node wants to send some packet. In other words, the discovery of the neighborhood is done only when the node wants to send a packet. Despite reducing overhead once the number of messages is reduced, this approach can degrade the network if its mobility is high, since the changes will only be noticed when the node needs to send a packet. The second method is called Event-Based Hello and the adjustment is made based on the events that occur in the network. In this approach, at first a network node sends Hello messages with the default frequency, but if after a predefined period of time that node does not receive any Hello messages from a neighbor or does not need to send packets it stops sending Hello messages. The problem with this approach is that if all the nodes in the network move away and after the time period return to get closer, no one will send Hello messages and the topology information would be out of step until a node decides to send a packet with the same problem as the Reactive Hello approach. In the third method, called Adaptive Hello, each node in the network sends a Hello after moving a defined distance. The problem with this algorithm is that each node needs to assume its position. In Reference [43], the frequency depends on the speed of the nodes, and the problem of this approach is when there are nodes that do not move but disconnect, for example, to save energy.

The works previously mentioned demonstrate that a dynamic adjustment reduces overhead in relation to the simplistic model where the frequency of sending messages is defined in a static manner using fixed values. In this context, the goal is that the algorithm adjusts the sending of Hello messages according to the mobility of the network. The mobility occurs when a node moves out of the reach of neighbors, shuts down or it is inoperative. In case of mobility events, the frequency is adjusted to higher values so that the new network information can converge quickly. If there is no mobility on the network, the frequency should be reduced but not suspended as proposed in other works.

In this context, the main contributions of this paper can be summarized as follows:

- To develop an enhanced routing protocol based on RL technique, named e-RLRP, that is able to learn from network events history, avoiding paths with connection problems. Also, it is able to reduce the number of control messages. The routing algorithm based on RL is developed according to Reference [40].
- Implementation of an algorithm that compensates the overhead inserted by the messages related to RL algorithm in the RLRP. To the best of our knowledge, a dynamic adjustment algorithm of Hello Message time interval to compensate the overhead has not been treated by other routing protocols based on RL. Thus, the present research contributes with the advances in the state-of-the-art of these protocol types.
- The performance of the proposed method is compared to other widely used routing protocols, such as the Better Approach To Mobile Ad-hoc Networking (BATMAN) and Optimized Link State Routing (OLSR), and also the RLRP protocol. To this end, different network typologies and traffic flows were implemented. The performance comparison considers key network parameters, such as throughput, packet loss rate and delay. Also, the speech perceptual quality in a VoIP communication service is evaluated, in which two operation modes of the AMR-WB speech codec [44] are used.

The algorithm to compensate for the overhead caused by the use of RL is based on the reduction of the overhead generated by another control message, the Hello message, which is responsible for the dissemination of information about the neighborhood of each network node. A dynamic adjustment in the frequency of sending the Hello message is capable of reducing the global overhead. The algorithm proposed in this work adjusts the sending of Hello messages according to the mobility of the network. Thus, this work contributes in the improvement of routing protocols based on RL technique, because it addresses one of the deficiencies of these protocols, which is the increasing number of control messages; therefore, the network overhead is also affected.

In this work, different ad-hoc multihop network scenarios are implemented, considering different network topologies, a variable number of nodes, different traffic flows and several degrees of network mobility. In order to simulate network failures, some nodes drop in random instants during each simulation. In these scenarios, a VoIP traffic is simulated and used as a case study. To this end, an UDP traffic is defined between a pair of source and destination nodes, and some nodes in the network are randomly turned off in order to simulate a network failure. Thus, it is possible to obtain network parameters, such as throughput, delays, packet loss rate and number of control message sent to the network, which are used to evaluate the impact of the routing algorithm on the perceptual quality of VoIP communication according to the E-model algorithm described in ITU-T recommendation G.107.1 [16]. It is important to note that VoIP service is used as a specific study case, but the proposed routing algorithm is for general purposes being agnostic of the service application. Finally, experimental performance results show that the proposed e-RLP overcame, in most of the test scenarios used in this work, the other routing protocols used for comparison purposes. The e-RLRP provides an overhead reduction of up to 18% compared to RLRP. The case study demonstrates that e-RLRP can provide a VoIP communication quality improvement of more than 90% if compared to OLSR, and up to 8% if compared to RLRP.

The remainder of this paper is structured as follows. In the Section 2 a theoretical review is presented. The proposed routing algorithm based on RL is described in Section 3. In Section 4, the different steps of the experimental setup are described. Section 5 presents the experimental results. Finally, the conclusions are presented in Section 6.

## 2. Theoretical Review

### 2.1. Reinforcement Learning

RL is a Machine Learning technique in which there is an agent that interacts with the environment through actions and receives rewards for the actions taken. The RL problem can be summarized as, an agent interacting with an environment in order to maximize the accumulated reward over time [35].

**Figure 1:**
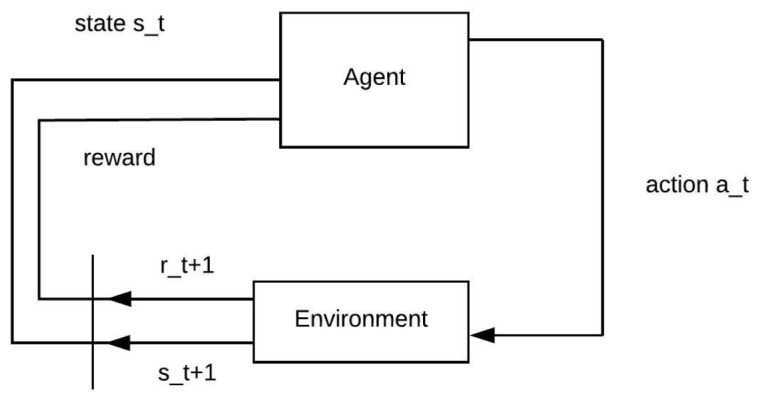
Generalized Reinforcement Learning Scheme.

The generalization of the RL interaction process [35] is shown in Figure 1, where the Agent interacts with the Environment through an $a_{t}$ action. This interaction leads to a new $s_{t+1}$ state and generates a reward for the Agent.

Through the rewards the agent estimates the action taken, and this knowledge is then used by the agent to adapt future decisions, which is usually controlled by the estimated value *Q*. In general, the estimated value defines how good a given action is.

The formulation of the RL optimization process can be represented as a Markov Decision Process (MDP) [35], introducing 4 sets S,A,P,R, where, *S* is a set of possible states of the agent; *A* represents the set of possible actions that an agent can take; *P* is defined as set of probabilities, in that an agent in a state *s*, advances to a state $s^{\prime }$ when opting for an action *A*. And finally the reward function *R*, which generates the reinforcement that the agent receives for choosing action *A*.

According to MDP the transition probabilities from *s* to $s^{\prime }$ after taking action *a* ($P{{}_{ss^{\prime }}}^{a}$), can be described as follows:

(1) $$P{{}_{ss^{\prime }}}^{a}=Pr\left\{s_{t+1}=s^{\prime }|s_{t}=s,a_{t}=a\right\}.$$

The estimation reward values (*E*) for action *a* ($R{{}_{ss^{\prime }}}^{a}$), from state *s* to $s^{\prime }$, is defined by:

(2) $$R{{}_{ss^{\prime }}}^{a}=E\left\{r_{t+1}|s_{t}=s,a_{t}=a,s_{t+1}\;=\;{}_{s^{\prime }}\right\}.$$

The sets *S* and *P* can be defined by a set of estimation values *Q*, which is dependent on the reward value obtained from an environment, and also from the current moment *t*, when the corresponding action has been performed. The estimation function values are presented as follows:

(3) $$Q_{k+1}=Q_{k}+\alpha *\left[r_{k+1}-Q_{k}\right],$$

where $Q_{k}$ represents the estimation value on the previous step; $Q_{k+1}$ is the current estimation value; $r_{k+1}$ define the reward value for an action performed on the current step; α represents step size parameter; and *k* is the current step number.

One of the main questions about RL is to take advantage of the current actions that generate greater rewards or explore new actions in order to be able to obtain even better rewards. In order to maximize the rewards received, the agent must balance the need to explore new actions or take advantage of current ones.

In RL, the most common methods for action selection are greedy, e-greedy and softmax methods [45]. The greedy selection of the action with the maximum estimation value all the time. The e-greedy selection of action with the maximum estimation almost every time, however, sometimes it explores new actions at random. The Softmax method [46] provides a dynamic change of selection probabilities of the actions. This change of selection probabilities of the actions occurs according to a predefined probability function, such as the Gibbs-Boltzmann distribution [35].

### 2.2. Ad-Hoc Networks Routing Protocols

The purpose of a routing algorithm is to find good paths between a source and a destination node. Usually, the best path is one that has the lowest cost [47]. There are several routing algorithms, some of them aim to find the lowest cost path according to a defined metric. Then, for these protocols, the most common metric is the hop count where the cost of a path is the sum of the number of hops between source and destination.

Ad-hoc network routing protocols must be able to handle a dynamic topology. This feature brings several challenges in their development. In general, these routing protocols can be divided into three subclasses:

- Reactive protocols [48] which exchange topology information on demand. In this type of protocol, the exchange of information about the topology occurs only when a node wants to send a message. In the reactive protocol, the redundancy in the transmission of service messages is lower in relation to other type of protocols.
- Proactive protocols [49] which continuously update the route information by sending control messages. In this type of protocol, the exchange of information between nodes about the network topology occurs even before any node sends any packets. Proactive protocols generally provide greater flexibility in route selection compared to reactive ones. However, it produces a greater number of control messages that increases the overhead.
- Hybrid protocols [50] that combine proactive and reactive characteristics. In hybrid protocols, some routes are created using a proactive approach and later the protocol works reactivelly.

As previously stated, for performance validation, the proposed algorithm is compared to BATMAN, OSPF and RLRP routing protocols, which are described in the following lines.

#### 2.2.1. Better Approach to Mobile Ad-Hoc Networking (BATMAN)

The BATMAN [51] is a proactive routing protocol for Ad-hoc network. It uses a different approach for sharing the knowledge about the best paths. Basically, each node has information about which jump distance neighbor has the best route to a given destination X, that is, which neighbor must be chosen when it is desired to send a packet to node X.

In the BATMAN each node in the network sends a message, called OriGinator Messages (OGMs), to all its neighbors to inform of its existence. The OGMs are small messages that contain the address of the originating node, the address of the node that relayed, a Time To Live (TTL) and a sequence number to record the route already taken by the packet. When a node receives an OGM message it updates its routing table, decreases the TTL and increases the sequence field. After that it forwards the message to its neighbors; this procedure is repeated until all nodes in the network receive the message.

BATMAN uses the exchange of OGMs messages to influence the choice of routes, basically this happens as follows: When an X node in the network receives the same OGM from a Y emitter through two different paths it discards the last message and considers only the first message. The idea is that OGM that arrived first probably traveled the best route.

The node X then records which neighbor of a jump emitted the OGM that arrived first. This neighbor is defined as the best path for a possible route to the Y transmitter. When OGMs go through bad routes are usually lost or take a longer time to arrive, thus, the node will only consider OGMs from good routes, that is, only the routes considered the best are recorded.

Another important mechanism of BATMAN is the selective flooding system that works as follows: When a node receives an OGM in addition to relaying the OGM received to neighbors it also responds the source node with another OGM message. However, it does not send the message in brodcast, it first queries in its table which neighbor has the best route to the source node and sends only to this neighbor. In this way, messages are sent selectively. Which decreases the overhead of control messages.

BATMAN is used as a reference in this work because it is a well-known protocol for ad-hoc networks.

#### 2.2.2. Optimized Link State Routing (OLSR)

The OLSR [52] is a proactive protocol commonly used in ad-hoc networks. The OLSR uses two control messages for topology discovery and maintenance: Hello and Topology Control (TC). Hello messages are used for neighbor discovery. The TC messages are used to disseminate information about neighbors and the state of the links established between them in order to build the network topology.

The OLSR employs a technique called Multi-Point Replaying (MPR) to reduce overhead caused by sending control messages and the number of rebroadcasting nodes [53]. This technique is to limit the number of neighbors that can relay control messages. For this to occur each node selects a number of neighbors that can relay the messages. Unselected neighbors receive the messages but do not forward them to other nodes. Additionally, TC packets include a sequence number to avoid infinite retransmissions due to undesirable loops.

The OLSR is used as a reference in this work because it is a widely used protocol in ad-hoc networks. In addition, this protocol provides better results, in terms of Quality of Service, than other routing algorithms [54] also considering the VoIP service [55].

### 2.3. Reinforcement Learning Routing Protocol (RLRP)

The RLRP is a reactive routing protocol for multi-hop ad-hoc networks. In general, the purpose of RLRP is to make a decision about the forwarding of packets to neighboring nodes based on estimated values. These values are dynamically updated through the rewards mechanism used by RL. The RLRP works on Linux systems with the TCP/IP stack providing routing for any data packets with either IPv4 or IPv6 addressing [40]. The routing process starts after initializing the routing daemon and runs on a created virtual interface.

The RLRP as any other conventional protocol for ad-hoc multi-hop networks is based on two operational modes. The first one is path discovery, which occurs when a node needs to send a packet and has no routing information for a destination. The second is packet forwarding, which is when a protocol decides which route is the best to send the packet [40].

In the first mode, the RLRP uses the reactive approach. Thus, a source node (A) sends a route request (RREQ) message to its direct neighbors and the neighbors in turn relay this RREQ to their neighbors. This manner, the RREQ message is forwarded to all network nodes until the transmission time-to-live (TTL) counter is reached or until a node that has already sent this RREQ receives the message again. All network nodes that participated in the RREQ relay get route information toward the source node and update their routing tables with that information. The destination node (B) receiving RREQ sends a route response message (RREP) that goes through the same relay process. Neighbor nodes of B and all node participating in RREP relay update their routing table with path information to reach node B. When the node A receives the RREP sent by the node B, all network nodes are already aware of the routes between A and B. Thus, the path discovery process ends and packet forwarding mode can be started.

Conventional routing protocols have in their routing table a field with destination address information. Each route is associated to a cost that is calculated according to an specific metric, then the path which has the lowest cost is selected. In turn, RLRP uses RL to decide which path is the best.

As explained in the Section 2.1 in RL there is an agent, a set of actions that the agent can do. Each agent’s action generates a reward. To this end, there is a set of estimations for the actions. For better association Table 1 introduces a relationship between reinforcement learning and conventional routing protocols.

The Table 1 shows the relationship between the routing mechanism/tasks and the Reinforcement Learning mechanisms. Through this relationship it is possible to apply the RL to the routing task. Thus, an X node of the network that uses the RLRP protocol can be considered an Agent.

The set of actions is the set of nodes in the network on which X can send messages. Sending a packet to a given network node is an Agent Action. And when sending this packet, node X expects to receive an Acknowledgment Message (ACK), if this happens it means that the message reached the given node, that is, a reward was generated for having chosen this node to send the message. If the ACK is not received it means that the message has been lost and the route is bad; then, node X receives a punishment for the chosen action.

Finally, the protocol routing table defines the best route to send a packet to a given destination. Similarly, an estimate set defines which action generates the best reward.

### 2.4. Speech Quality Assessment in VoIP Services

One of the major concerns in VoIP service is the cost associated with the transmission medium. Due to this fact, in a VoIP communication compression techniques are used, and they do not cause significant losses in the received signal quality [56]. Speech codecs are responsible for this compression. There are different speech codecs, one of the most adopted in current communication networks is the Adaptive Multi-Rate Wideband (AMR-WB) codec [57].

The AMR-WB is a speech codec used for mobile device communications. It is widely used by network operators to provide high quality conversations. AMR-WB is based on the linear prediction generated by the ACELP algebraic code that uses a vector quantization technique [58]. The AMR-WB uses nine operation modes, from 6.6 kbps to 23.85 kbps, and each of one has a different response to packet losses.

The *WB* E-Model algorithm [16] is a parametric method that predict a conversation quality using different impairment factors related to acoustic environment, network, and speech codec. The $R_{WB}$ is the global quality rating that is obtained using all the impairment factors. This value is expressed on a quality scale from 0 to 129, the higher the value the better the quality. The $R_{WB}$ score is determined by:

(4) $$R_{WB}=R_{0,WB}-I_{s,WB}-I_{d,WB}-I_{\mathrm{e-eff},WB}+A,$$

where $R_{0,WB}$ represents the basic signal-to-noise ratio (SNR), and for *WB* networks the standardized value is 129; $I_{s,WB}$ represents the combination of all impairments which occur simultaneously with the voice signal, for *WB* signals the adopted values of this factor is 0; $I_{d,WB}$ represents the impairments caused by delay and; $I_{\mathrm{e-eff},WB}$ is the quality degradation due to equipment, specifically the speech code; A represents an advantage factor, but in *WB* E-model is not considered and it is equal to 0. In this paper, we mainly focus on $I_{\mathrm{e-eff},WB}$, and the $I_{d,WB}$ is also evaluated.

The $I_{\mathrm{e-eff}}$ is determined by:

(5) $$I_{e,\mathrm{eff},WB}=I_{e,WB}+\left(95-I_{e,WB}\right)\cdot \frac{Ppl}{Ppl+Bpl_{WB}},$$

where $I_{e,WB}$ is the equipment impairment factor at zero packet-loss, only related to codec impairment; Ppl is the probability of packet losses, and the $Bpl_{WB}$ is the packet-loss robustness factor for a specific codec in *WB* networks.

In Annex IV of ITU-T recommendation G.113 [59], Ie and Bpl values for AMR-WB cocec are defined. Table 2 presents the number of bits and bit-rate of each AMR-WB operation modes, and their existing standardized $I_{e}$ and Bpl values. Note that some Bpl values are not defined (ND) in some cases.

In this work, the operation modes used in the simulation tests were 2 and 8, because they have the *Bpl* parameter already defined, as can be observed in Table 2. Thus, the $I_{e,\mathrm{eff},WB}$ value can be computed.

The $I_{d}$ is computed using the following relation:

(6) $$I_{d,WB}=I_{\mathrm{dte},WB}+I_{\mathrm{dle},WB}+I_{\mathrm{dd},WB},$$

where $I_{\mathrm{dte},WB}$ gives an estimate for the impairments due to talker echo, $I_{\mathrm{dle},WB}$ represents impairments due to listener echo, and $I_{\mathrm{dd},WB}$ represents the impairment caused by an absolute delay $T_{a}$ in the network. In this study, the $I_{\mathrm{dte},WB}$ and $I_{\mathrm{dle},WB}$ are not considered because they are related to echo and acoustic problems at the end-sides of the communication that is out of the scope of this research.

The $I_{\mathrm{dd},WB}$ is defined by:

(7) $$I_{\mathrm{dd},WB}=\left\{\begin{array}{c} 1,\;T_{a}<100\;\mathrm{ms}\\ 25\left[{\left(1+X^{6}\right)}^{\left(\frac{1}{6}\right)}-3{\left(1+{\left(\frac{X}{3}\right)}^{6}\right)}^{\frac{1}{6}}+2\right],\;T_{a}>100\;\mathrm{ms} \end{array}\right.$$

where

(8) $$X=\frac{\log(\frac{T_{a}}{100})}{\log2}.$$

It is important to note that in the proposed network scenarios, the Ppl and $T_{a}$ variable values can be obtained in each simulation test; therefore, the $R_{WB}$ can be computed using (4).

## 3. The Proposed e-RLRP Algorithm

In this section, the proposed e-RLRP algorithm is explained. Firstly, the RL technique in the routing protocol is implemented according to Reference [40]. Later, the proposed method to reduce the overhead is detailed.

### 3.1. Reinforcement Learning Used in Routing Protocol

The reward propagation with Acknowledgment message, the reward generation and the estimation values are presented.

#### 3.1.1. Reward Propagation with Acknowledgment Message (ACK)

The reward value is directly related to the receipt of the ACK. When a node wants to send a packet to a given destination it selects a neighbor from the existing one and sends the packet to that neighbor. After that, it waits for the corresponding ACK message, which contains meta-information about the received packet, and the reward value by the action of choosing this neighbor. This ACK message can return using a path different from the one used to send the corresponding packet.

If the ACK is not received within a pre-defined time then the sender node sets a punishment, that is, a negative reward to the neighboring node to which the packet was forwarded. This negative value is set to −1. If the ACK is not being received probably the neighboring node has gone offline. The neighboring may be experiencing hardware issues such as power outages, strong interference with wireless transmission or the node is overloaded with incoming traffic. Hence, it is consistent that this neighbor should be avoided in the future.

If the ACK message is received on time a reward value will be provided within the message. If the value is high it means that the neighbor has a good way to the destination, the probability of choosing this neighbor in the future will increase. If the value is low it means that the chosen neighbor does not have a good route to that destination, because it has hardware problems, there may be many hops or the further links quality is weak. In this case the source node will slowly decrease the estimation value for this neighbor, which is likely to cause the node to later choose other neighbors.

#### 3.1.2. Reward Generation

The mechanism for adjusting the reward value must be flexible, that is, the adjustment may not be too small that do not cause changes or too large as to induce sudden change due to a specific events. For example, if the value of the punishment after choosing a bad route is too low, the estimated value of that route will slowly decrease and probably this bad route can still be chosen for a long time. On the other hand, if the punishment value is too high, a route may no longer be chosen because of just one packet loss event. Therefore, a balance must be found between low and high rewards/punishment.

According to Reference [40], the reward value is calculated as follows: When a node X receives a packet of node Y, an ACK is sent with the reward value to Y. To calculate the reward value, the sum of the estimated values that each neighbor has in relation to destination node Y, called $Q_{{\mathrm{dst}}_{\mathrm{ip}}}$ is divided by the corresponding number of neighbors (N). Thus, the ${\mathrm{reward}}_{\mathrm{value}}$ is the average of the *Q* values of the neighbors in relation to node Y. The ${\mathrm{reward}}_{\mathrm{value}}$ is calculated according to:

(9) $${\mathrm{reward}}_{\mathrm{value}}=\sum Q_{{\mathrm{dst}}_{\mathrm{ip}}}/N.$$

Upon receiving the ${\mathrm{reward}}_{\mathrm{value}}$, the node Y adjusts the estimated value for node X. However if the ACK is not received, the node Y automatically set the reward value to −1, that is, a punishment is generated that negatively impacts the estimated value for the route. The estimation value is defined in the next subsection.

#### 3.1.3. Estimation Values Based on Rewards

An initial value must be set for each node when the protocol starts, which is often called cold start. The RLRP initially defines all neighbors with a value of 0 when a source node has no route information towards a destination node. The available range of estimated values is defined as: [0, 100]. When the protocol starts the route discovery process the estimate values are set as follows:

(10) $$Q_{n}=100/N_{\mathrm{hops}},$$

where $Q_{n}$ is the estimated value for destination IP towards neighbor n; $N_{\mathrm{hops}}$ is the number of hops in which RREQ or RREP messages has traversed from the source to the destination node

After the path discovery procedure ends all nodes in the network have the initial estimated values for all routes. According to the calculation presented in (10), the estimation value is initially defined based on the number of hops between the source and the destination. It can be defined that the RLRP uses an initial approach of the hop count metric, in which the routes with the least hop are chosen.

However, afterwards the values be adjusted since the route with the least number of hops is not always the best one. For, a route may have the least number of hops but present an overloaded link or have nodes that present malfunctions. The adjustment is made according to the received reward value. The estimation value *Q* like described in Section 2.1 is calculated as follows:

(11) $$Q_{k+1}=Q_{k}+\alpha *\left[r_{k+1}-Q_{k}\right],$$

where $Q_{k+1}$ represents the new estimation value for the action; $Q_{k}$ is the actual estimate value; $r_{k+1}$ define the reward value obtained; α represents step size parameter; and *k* is the current step number. Therefore, the estimated value as stated above is impacted by the reward value.

In e-RLRP, the reward is associated with the successful delivery of packets. Then, in general, the local reward is given to the route that has the best rate of success in delivering packets, and the long-term reward is related to the global network performance by always looking for routes with the highest success rates. As explained in Section 2.1, the RL algorithm has to consider two approaches in order to obtain a long-term reward, the selection of actions that obtain the highest reward values or explore new actions that can generate even better rewards. For this decision task, the e-RLRP uses the Softmax method [60].

### 3.2. Algorithm Used in the e-RLRP to Reduce the Overhead

To send a packet, the node needs to know what neighbors nodes are directly connected. Therefore, a neighborhood discovery procedure is required. In RLRP, this procedure occurs through the broadcasting of messages called Hello.

By default, Hello messages are sent every 2 s, thus, the information about neighbors is updated in the same period of time. This update interval parameter is called Broadcast Interval (BI). The RLRP has 10 types of headers, two of them are Reward Header and Hello Header. The structure of data fields of the Reward Header and Hello Header are shown in Table 3 and Table 4, respectively.

The Reward Header is 8 bytes. The Type field defines what the header is, the ID field is the unique identifier of the message service. The Neg Reward Flag field is a test flag that checks whether the reward is negative or positive, the Reward Value is a value of reward, and finally, the Msg Hash is the identifier of the packet to which the reward belongs.

The Hello Header size ranges from 4 to 56 bytes, this variation depends on the node address that can be IPV4 or IPV6. The Type field defines what the header is, the field IPv4 Count defines the number of assigned IPv4 addresses, limited to one. The IPv6 Count is number of assigned IPv6 addresses, limited to three. Tx Count is the number of re-broadcasts, GW Mode define that a node is a Gateway in the network, the IPv4 and IPv6 address define the address of the node.

As can be observed in Table 3, the Reward header used in RLRP is 8 bytes long and generates an additional overhead, which corresponds to the use of RL technique.

In this context, the present research implemented an algorithm to reduce the overhead generated by the Hello message, specifically to reduce the frequency of sending Hello messages in order to compensate the additional overhead generated by the Reward header.

It is clear that increasing the time interval for sending Hello messages, defined by the *BI* parameter, will decrease the frequency of sending messages and consequently, the overhead is also decreased. However, a high value also impacts the time of updating information about the neighborhood, and the routing can be negatively affected.

Thus, the proposed algorithm implemented in the e-RLRP is capable of dynamically adjusts the frequency of sending Hello messages. This adjustment in the parameter *BI* is made according to the mobility present in the network. If the network is static, that is, no neighbors enter or leave the coverage range, it is not necessary to send Hello messages with a high frequency. Otherwise, if the network presents a high mobility, to send messages more frequently is necessary.

**Figure 2:**
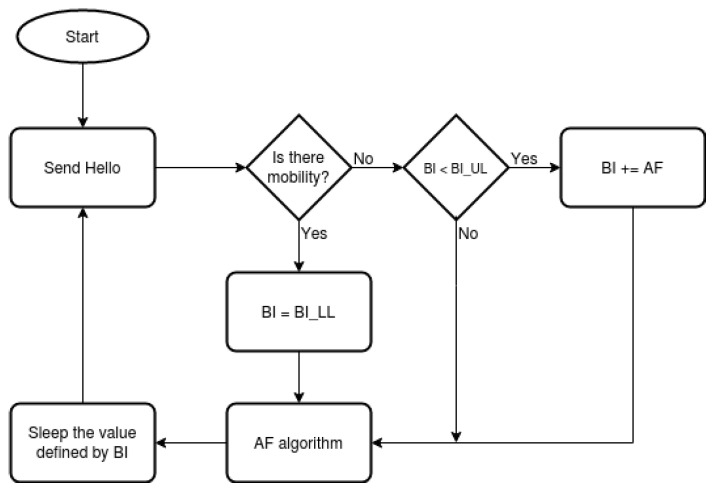
Scheme of the dynamic adjustment algorithm proposed.

A general high representation of the proposed algorithm is introduced in Figure 2.

The sending of Hello messages starts together with the e-RLRP daemon. Next, the algorithm checks the mobility of the network. To this end, there is a function named Update Neighbors File responsible for updating the list of neighbors every time a Hello from a new node is received. And there is a other function named Check Expired Neighbors that checks if a Hello message has been received from neighbors every 7 s, if a neighbor is 7 s or more without sending a Hello, it is removed from the list because it is out of reach. This interval of time was defined experimentally in Reference [40]. In case, a new neighbor is detected or an existing one is lost, it will be considered that there is mobility in the network.

In the proposed e-RLRP, when mobility occurs, the *BI* value will be reduced to a lower limit called *BI* Lower Limit ($BI_{\mathrm{LL}}$), the algorithm waits for a new time interval, sends a message and restarts the process. If a mobility event does not occur, the *BI* parameter will be increased with the Adjustment Factor (*AF*) parameter respecting the upper limit called *BI* Upper Limit ($BI_{\mathrm{UL}}$). When the time defined by *BI* is reached, a Hello message will be sent and the process is restarted. Hence, the frequency of sending Hello messages is adjusted according to the mobility of the network

It is worth mentioning that the proposed dynamic adjustment is not based on RL, because RL uses more computational resources.

#### 3.2.1. Definition of the Upper and Lower Limits of Broadcast Interval

The higher the value of the *BI* parameter, the lower the frequency of sending Hello messages, and consequently the overhead is reduced. However, it is necessary to define a limit to that value does not grow indefinitely.

The $BI_{\mathrm{UL}}$ cannot be greater or equal than 7 s due to the Check Expired Neighbors function. Otherwise, the network nodes will be eliminated when the function timeout will be reached. Considering that the $BI_{\mathrm{UL}}$ value must be lower than 7 s and also the latency of the existing network, the value 6 s is defined in order to guarantee that neighbors are not erroneously removed.

To define the $BI_{\mathrm{LL}}$, 3 values of *BI* lower that 2 s are tested in the scenario called Programmed that is described in Section 4.1. The overhead is calculated considering the source and destination node. The *BI* value of 2 s, defined in the RLRP, is also tested in the same scenario, and the overhead obtained was 1.42 MB. The *BI* values 0.5, 1.0 and 1.5 were tested. Table 5 shows the overhead results for each *BI* value.

Table 5 shows the increase in overhead of the tested values in relation to the default value used in RLRP. The *BI* value of 1.5 had a gain of 2.12%, the value of 1.0 presented an increase of 4.22%. The value of 0.5 obtained the highest increase, a gain of approximately 12.67%. Considering this value as a high increase in overhead compared to the previous ones, the value of 0.5 is discarded. Hence, we opted for the intermediate tested value, and $BI_{\mathrm{LL}}$ is set to 1.0.

#### 3.2.2. Adjustment Factor

The objective of the e-RLRP is to reduce overhead but without degrading the performance of the algorithm. Thus, after a scenario of high mobility is detected, the rise of the *BI* parameter should be slower to ensure that the upper limit is slowly reached, because there is a likelihood that the occurrence of mobility will repeat itself. In a scenario in which an isolated episode of mobility occurs, the climb should be a little faster. Therefore, the *AF* also should has responses according to the mobility of the network. It is important to note that in initial tests, we used fixed values for the frequency of Hello messages, and the results demonstrated that dynamic methods permit to obtain better results in terms of the network performance parameters used in this work.

To ensure that no sudden changes occur in the *AF*, a scale of ten positions is defined, in which the upper limit is called $AF_{\mathrm{ul}}$ and the minimum value is called $AF_{\mathrm{ll}}$.

Also in the Programmed scenario described in the Section 4.1, the convergence time (*CT*) of the e-RLRP, which is defined as the time elapsed between breaking a route until the algorithm converged to find a new route, was also evaluated. Experimental test results demonstrated that the average of *CT* is 20.6 s.

The *AF* value cannot be high to avoid be aggressive enough to *BI* parameter reach the $BI_{\mathrm{UL}}$ before the *CT*. Then, to calculate $AF_{\mathrm{ul}}$ the Arithmetic Progression (AP) or also known as arithmetic sequence is applied, with a difference between the consecutive terms equal to BI, where term $A_{1}$ is $BI_{\mathrm{LL}}$, $A_{n}$ is $BI_{\mathrm{UL}}$ and the sum of the terms must not be greater than CT.

To ensure that value is not reached before 20.6 s, the value is rounded to 21 s and applying the formula of the sum of a AP, the $AF_{\mathrm{ul}}$ value is obtained.

(12) $$CT\leq \frac{({BI}_{\mathrm{UL}}+{BI}_{\mathrm{LL}})\times n}{2}.$$

Applying the result of Equation (12) in the formula for the general term of a AP:

(13) $${BI}_{\mathrm{UL}}={BI}_{\mathrm{LL}}+\left(n-1\right)\times {AF}_{\mathrm{ul}}.$$

The value obtained for $AF_{\mathrm{ul}}$ is 1, then, the maximum value of *AF* should be 1. As previously stated, a scale of 10 position was defined. Then, the value of $AF_{\mathrm{ll}}$ is 0.1, and each position of that scale is increased by 0.1.

Whenever mobility occurs in the network the *AF* is decreased in the scale. The increase will occur when there is a tendency of decrease in mobility during a period of time.

This period of time called Time of Check (*TC*) is defined by the average between *CT* value and the time spent for the algorithm starting from $BI_{\mathrm{LL}}$ until reaching value $BI_{\mathrm{UL}}$ with adjustment $AF_{\mathrm{ll}}$. To calculate *TC*, first, the formula of the general term of AP is applied. The $AF_{\mathrm{ll}}$ is the common difference, $BI_{\mathrm{UL}}$ and $BI_{\mathrm{LL}}$ are the terms An and A1 respectively.

(14) $$BI_{\mathrm{UL}}=BI_{\mathrm{LL}}+\left(n-1\right)*AF_{\mathrm{ll}}.$$

Applying the result of Equation (14) in the formula for the sum of a AP and averaging:

(15) $$TC=\frac{CT+\frac{(BI_{\mathrm{LL}}+BI_{\mathrm{UL}})*n}{2}}{2}.$$

The *TC* value is 99.7, in this way after 99.7 s if there is a tendency to reduce mobility, the *AF* will be increased. A mobility counter denominated $M_{\mathrm{counter}}$ will be used to count how many mobility events occur in the *TC* time period. Whether when a new neighbor comes within range of a given node or when a neighbor leaves within range of that node

(16) $$M_{\mathrm{counter}}=NewNeighbors_{\mathrm{counter}}+LostNeighbors_{\mathrm{counter}}.$$

Belonging to a family of statistical approaches used to analyze time series data in the area of finance and technical analysis [61], the Exponential Moving Average (*EMA*) can be used to estimate values [61,62,63]. The *EMA* is used to calculate if the occurrence of mobility tends to increase or decrease according to the Equation (17). The *EMA* is applied in a series of 10 values $M_{\mathrm{counter}}$.

(17) $$EMA_{k}=\left(M_{\mathrm{counter}}-EMA_{k-1}\right)\left(\frac{2}{\left(N+1\right)}\right)+EMA_{k-1}.$$

If $EMA_{k}$ < $EMA_{k-1}$, the number of mobility events has a tendency to decrease, then the *AF* value will be increased. The period *N* of 10 values was chosen precisely because it is the number of times that *AF* must be increased until reaching $AF_{\mathrm{ul}}$.

**Figure 3:**
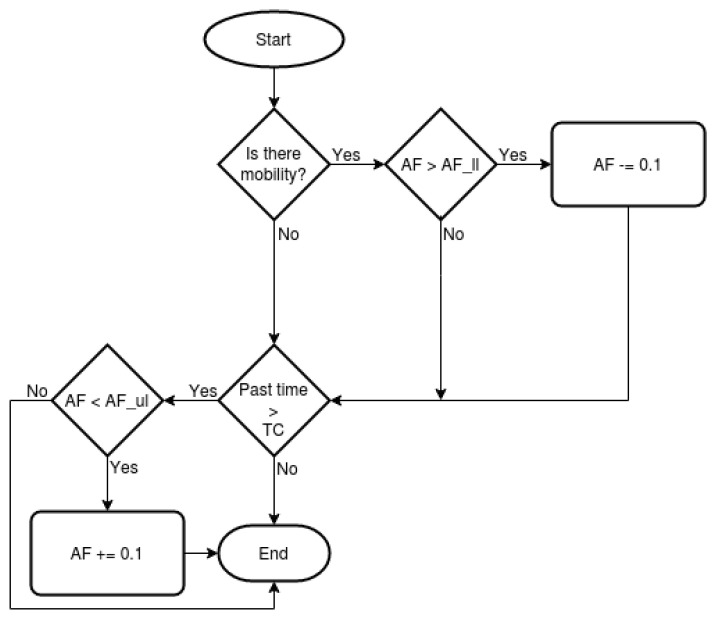
Scheme of dynamic adjustment of Adjustment Factor (*AF*).

The scheme of the *AF* adjustment algorithm is shown in Figure 3.

Thus, the *BI* is adjusted according to the mobility of the network, making possible to reduce overhead.

## 4. Experimental Setup

In this section, different network scenario configurations used in the simulation tests for performance validation of the proposed e-RLRP are described. Different network topologies with different numbers of nodes, routes, traffic flows and network mobility conditions are considered. Firstly, the four network topologies used in the simulations are described. Later, two simulation scenarios are explained. Finally, the transmission rate in the scenarios are explained, and the simulation environment is described.

### 4.1. Network Topology

**Figure 4:**
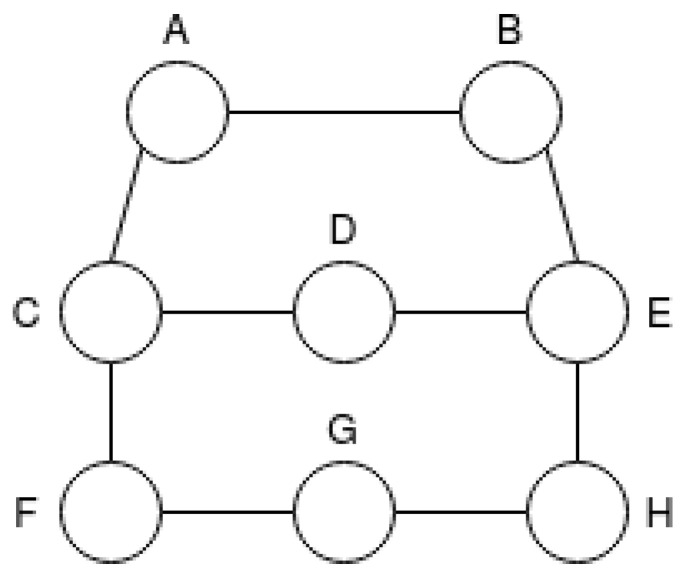
Network Scenario Topology T1.

In this work, four network topologies were created to simulate a wireless node network, which are called T1, T2, T3 and T4. Node names were distributed in order to improve the understanding of the scenarios that will be described later. The topologies were developed in order to guarantee that each route has a different number of hops. In Topology T1, there are 3 routes and 8 nodes as illustrated in Figure 4.

**Figure 5:**
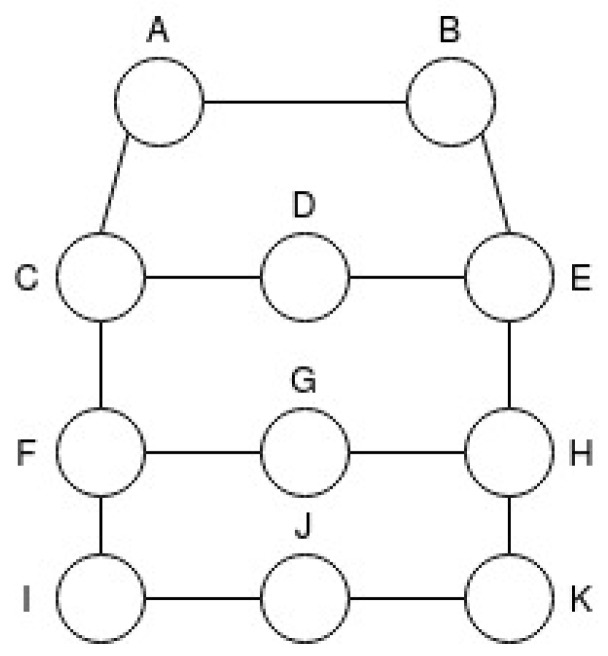
Network Scenario Topology T2.

The Topology T2 is an extension of T1 with the addition of three nodes, thus, in total there are 11 nodes and four different routes, which are distributed according to Figure 5.

**Figure 6:**
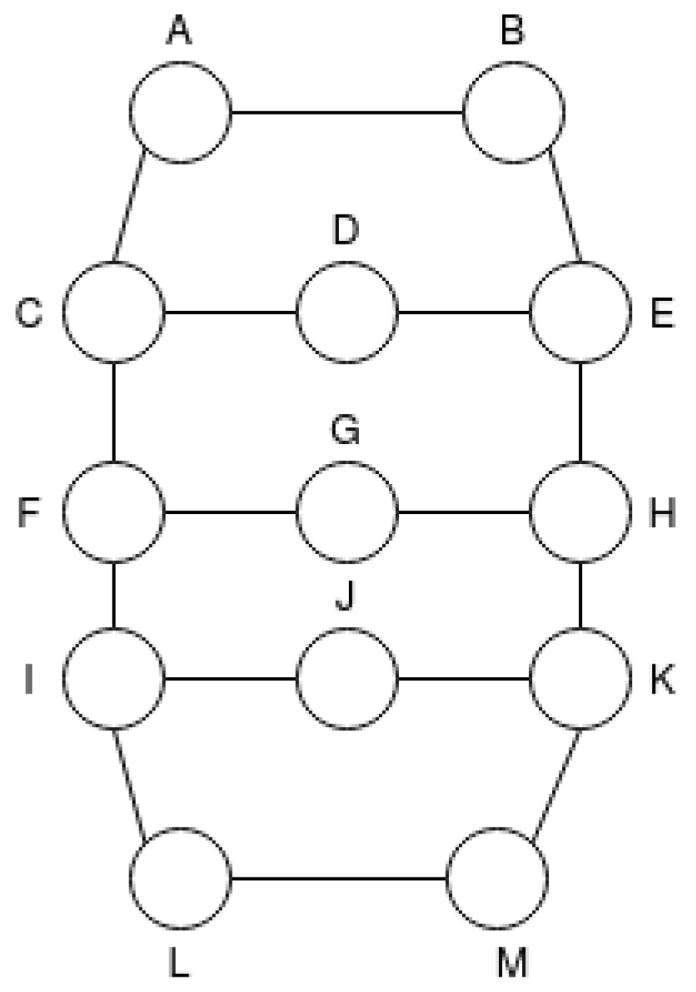
Network Scenario Topology T3.

The T3 is illustrated in Figure 6. This topology is also an extension of the T1 but now we add five extra nodes; thus, there are a total of 13 nodes with 5 different routes.

**Figure 7:**
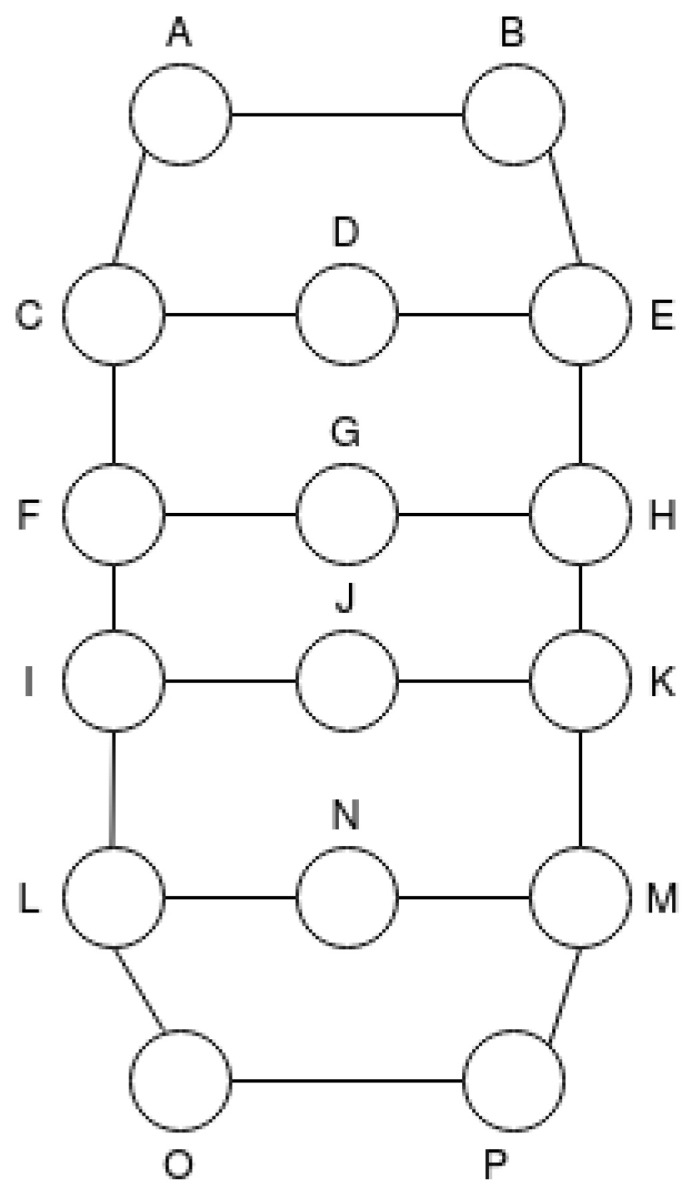
Network Scenario Topology T4.

The T4 is illustrated in Figure 7. This topology like the others is an extension of the T1 but now we add eight extra nodes; thus, there are a total of 16 nodes with 6 different routes.

### 4.2. Emulation Scenario

In order to test the functionalities of the e-RLRP, two different scenarios were developed in which there are routes that degrade network performance. To this end, some nodes in the network were configured to disconnect on a recurring basis at random instants, simulating node failures and mobility in the network.

In the first scenario, only topology T1 is used. A flow is defined with node C being the source and E being the destination, the node D will be programmed to shut down 5 times. This node is part of the shortest route between the source and destination of traffic for T1. For a better later association, the first scenario is named Programmed (P). Thus, the scenario P is a proof of concept to test the RL in the e-RLRP, in which a better performance than other protocol is expected. In principle, the route with the least number of hops is the best path and is the one that should be chosen initially by all protocols. However, in this scenario, the choice of this path will cause degradation in the network since there is a node that recurrently disconnects causing packet loss. As the e-RLRP can learn from the network, it should be able to avoid the path containing nodes which present recurrent drops.

The second scenario, named Random (R), also a flow is defined with node C being the source and E being the destination. The nodes A, D and G of topology T1; nodes A, D, G and J of T2; nodes A, D, G, J and L of T3; and nodes A, D, G, J, N, and O of T4 are randomly disabled at different instants, in order to simulate random drops. In addition, 3 configurations for drops are defined. In the first configuration, 3 drops are drawn between the aforementioned nodes for each topology. In the second configuration, 5 drops are drawn, and in the third configuration 7 drops are considered. The reason for choosing only these nodes is to ensure that each route has only one node that fails, thus, the same probability to draw a drop for each route is ensured. These nodes are randomly disconnected in each simulation. The instants in which each node drops during the simulation is randomly defined, then, the routing algorithm does not know which node is down to avoid that path. The objective of this scenario is to test the e-RLRP in a random scenario when the network degradation increases. The network scenarios characteristics used in this research are different from network scenarios in which node drops are controlled, and a scheduler can be implemented in the network. It is important to note that the e-RLRP could also work in conjunction with a scheduler for more complex network scenarios, but these scenarios are out of the scope of this present research.

Additionally, two different configurations of the scenario R is defined for topologies T3 and T4 where the flow number is greater than one. A network configuration with 3 flows is defined, in which the first one is from node C to E, second one from node F to B and third one from node I to H. The second network configuration considers 4 flows, where an additional flow from node M to K is added to the three previous mentioned flows. The objective of these two scenarios, is to investigate the impact of the additional network overhead due to RL control messages.

In these both scenarios, the ability of e-RLRP to avoid routes that degrade the network through the use of RL is tested. And mainly the ability of e-RLRP to reduce network overhead in mobility scenarios is also evaluated, providing a higher throughput and reducing the *Ppl* value.

Table 6 shows which nodes have been configured to shut down simulating a drop in T1, T2, T3 and T4 topologies for all scenarios. In the Programmed Scenario uses only the T1 because it is a scenario for proof of concept.

### 4.3. Transmission Rates of AMR-WB Codec

This work also aims to test the impact of the previously mentioned routing protocols in a real communication service, to this end, VoIP communication scenario is used as a case study. Thus, a traffic from node C to E is simulated with different bit-rates defined according to the AMR-WB codec. In addition, we used UDP communication and a packet time-length of 20 ms.

Speech signal transmitted on an IP network is compressed by a speech codec, and them this payload must be packaged. For this, Real Time Protocol (RTP), the UDP and IP headers are inserted. The bit-rates presented in Table 2 only refer to the payload, then, it is necessary to add the number of bits regarding the RTP (12 bytes), UDP (8 bytes) and IP (20 bytes) headers to obtain the transmission rate. For example, AMR-WB-Mode 2 (12.65 kbps) contains 253 bits that are sent every 20 ms, then if the 320 bits of headers are added, a total of 573 bits are sent in this same period of time, that represents a transmission rate of 28.65 kbps. Table 7 shows the transmission rates used in the test scenarios.

### 4.4. Emulation Environment

To test and analyze the performance of the four protocols previously mentioned, we use the network emulator Common Open Research Emulator (CORE) [64]. Developed by Boeing’s Research and Technology division, CORE is a real-time, open source, emulator. The CORE is chosen because it enables the use of real-world routing protocols and applications using Linux system virtualization. The e-RLRP code must be executed on a Linux platform. Each node in the emulator is a virtual machine with network interface and resources shared with the host machine. The e-RLRP, RLRP, BATMAN and OLSR routing protocols are installed to be used by network nodes.

The network performance metrics obtained in the tests were throughput, *Probability of Packet Loss* (*Ppl*), the Round Trip Time (RTT) and Overhead. The throughput and *Ppl* values are calculated using Iperf tool [65]. It is capable of generating UDP and TCP traffic streams at defined rates. To calculate RTT, the UDP stream is replaced by an ICMP stream generated by the native Linux PING command. The PING command itself returns the RTT value. The Overhead is measured using the WireShark [66] tool. In addition to the aforementioned tools, the native Linux shell script is used to shutdown nodes on a programmed or random basis.

Finally, the speech quality of a VoIP communication is evaluated. To this end, the network parameters, such as *Ppl* and delay were used as inputs of the E-model algorithm to estimate the communication quality.

## 5. Results and Discussions

In order to evaluate the e-RLRP performance in relation to BATMAN, OLSR and RLRP protocols, different network scenarios were emulated. Each simulation scenario runs 50 times, and the average value for each scenario is computed. The simulation of each scenario takes 600 s.

In the test scenarios, the AMR-WB operation modes 2 and 8 were considered. Thus, the transmission bit-rates considered were those presented in Table 7.

Firstly, an ideal scenario without drops is tested to assess the overhead reduction obtained by the e-RLRP in relation to RLRP. The Table 8 shows the overhead in the network scenario without drops, these results represent the average overhead of the nodes, considering AMR-WB Modes 8 and 2.

As expected, the results obtained in the ideal scenario without drops demonstrate that e-RLRP obtained an overhead approximately 16% lower than RLRP. This result is due to the fact that the e-RLRP in a scenario without falls keeps the frequency of sending messages lower than the RLRP.

In a real ad-hoc network environment, nodes move or may fail, degrading the network performance. Therefore, the e-RLRP, RLRP, BATMAN and OLSR protocols are testing in scenario where mobility occurs. The throughput and *Ppl* results, in the so-called scenario P, are illustrated in Table 9 and Table 10, respectively.

Results presented in Table 9 and Table 10 demonstrate that e-RLRP and RLRP have a better performance than BATMAN and OSLR. The e-RLRP and RLRP have a *Ppl* value close to zero because they avoid the route containing node B that presents recurring drops. The value does not reach zero because when the routing starts, both protocols choose the route of node B which has the lowest number of hops, but after successive drops of node B, both protocols no longer consider the use of this route.

Differently, the OLSR protocol chooses the route which contains node B, because this is the path with the least number of hops. Despite the BATMAN protocol having obtained a higher *Ppl* than e-RLRP and RLRP, it presented a performance better than OLSR. This is due to the OGM messaging mechanism.

The overhead results for scenario P considering AMR-WB Modes 2 and 8 are shown in Table 11.

The overhead results presented in Table 11 show that the e-RLRP reduced the overhead in relation to the RLRP by approximately 7%, and also got better results than BATMAN and OLSR protocols. This happens because the e-RLRP reduced the frequency of sending Hello messages.

Similarly, the same network performance parameters are evaluated in R scenario. The Throughput, *Ppl* when nodes are shut down 3 times are presented in Table 12 and Table 13, respectively.

As can be observed in Table 12 and Table 13, the OLSR had the worst performance considering *Ppl* and Throughput, which is explained by the use of RL in e-RLRP and RLRP, and by the BATMAN OGM message mechanism. The e-RLRP reached similar throughput results to the other protocols, but in some scenarios, the *Ppl* had a significant reduction with e-RLRP.

The overhead when nodes are shut down 3 times are presented in Table 14. The results presented in the Table 14 demonstrate that the overhead of e-RLRP is lower than that of RLRP, reaching in some scenarios a reduction close to 18%.

The Throughput, *Ppl* and Overhead when nodes are shut down 5 times are presented in Table 15, Table 16 and Table 17, respectively.

According to the results obtained in a Five Drops scenario, the e-RLRP and RLRP algorithm performed better than BATMAN and OLSR. Also, the e-RLRP obtained an overhead reduction and *Ppl* lower values in relation to RLRP.

Similarly, the Throughput, *Ppl* and overhead, when nodes are shut down 7 times, are presented in Table 18, Table 19 and Table 20, respectively.

According to the presented results, the scenario where 7 drops occurs, the e-RLRP obtains better performance in all cases compared to BATMAN and OLSR, and also it presents a better performance than RLRP in most of the scenarios.

In general, the performance gain in scenarios R was lower than in scenario P. This behavior is because, in the scenario P the drops are recurrent in only one route, which facilitates the learning process of the e-RLRP.

**Figure 8:**
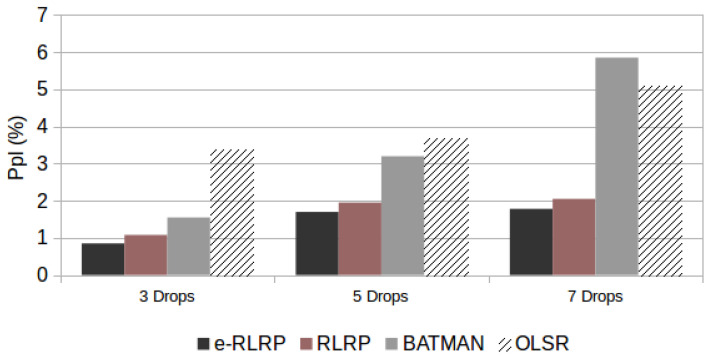
e-RLRP Performance, in terms of *Ppl*, and considering different number of drops.

Figure 8 demonstrates the e-RLRP performance improvement in relation to the other protocols. The *Ppl* values obtained is the average of the results obtained in the four topologies and both AMR-WB rates modes used in the tests.

From the results we can conclude that the performance of the e-RLRP in relation to the other three protocols increases when the number of drops increases. By increasing the number of drops, the performance of all algorithms degrades, however, in the e-RLRP and RLRP this degradation is lower.

**Figure 9:**
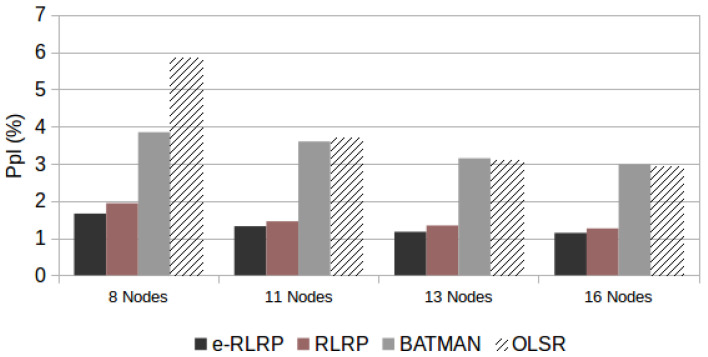
e-RLRP Performance, in terms of *Ppl*, and considering different number of nodes.

Figure 9 shows the relationship between e-RLRP performance and the number of nodes in the network. The *Ppl* values obtained are the average of the results obtained in the scenarios of 3, 5 and 7 drops and both AMR-WB rate modes used in the tests. It is important to note that the higher the number of nodes in the network, the higher the processing needed by the RL algorithm to determine the reward values. Despite the RL processing increases, the performance obtained by the e-RLRP, in terms of *Ppl*, is superior in relation to the other routing protocols.

The Throughput, *Ppl* and overhead, for three flows considering AMR-WB Modes 8, are presented in Table 21, Table 22 and Table 23, respectively.

Similarly, the Throughput, *Ppl* and overhead, for three flows, and considering AMR-WB Mode 2, are presented in Table 24, Table 25 and Table 26, respectively.

Analyzing the scenario with 3 flows, it can be seen that the e-RLRP overcomes in most cases the other protocols considering *Ppl* and Troughput. Regarding overhead, the e-RLRP reached the best results in all the network scenarios.

The Throughput, *Ppl* and overhead, for four flows, and considering AMR-WB Mode 8, are presented in Table 27, Table 28 and Table 29, respectively.

Similarly, the Throughput, *Ppl* and overhead, for four flows, and considering AMR-WB Mode 2, are presented in Table 30, Table 31 and Table 32, respectively.

We can see from the results of the 4-flow scenarios that e-RLRP outperforms other protocols in most cases in terms of Throughput and *Ppl*. Regarding the overhead e-RLRP obtained the best results in all tested scenarios. In addition, it is worth mentioning that the overhead in general increases when there are more flows, however, specifically the overhead generated by the Hello control message is not so impacted. This is because Hello messages are exchanged regardless of the number of streams in the network.

In the results presented in Table 27, Table 28, Table 30 and Table 31, regarding Throughput and *Ppl*, we can observe that one of the traffic flow (noted as MK) reached a *Ppl* almost equal to O, because there is a direct route between the two pairs of nodes and no drops occurred in this path. The extra flows were added in order to overload the network.

Analyzing the results of the scenarios with more than one traffic flow, specifically three and four flows, it is possible to observe that the e-RLRP outperforms the other routing protocols in most of the network scenarios tested, in terms of *Ppl* and Throughput. Regarding overhead, we can see that e-RLRP continues overcoming the other protocols. The experimental results confirmed that e-RLRP obtained a lower overhead than the RLRP in most of the scenarios, even when the number of traffic flows, the number of routes or node drops were increased. Thus, these demonstrated that the proposed adjustment function worked properly in the task of overhead reduction.

**Figure 10:**
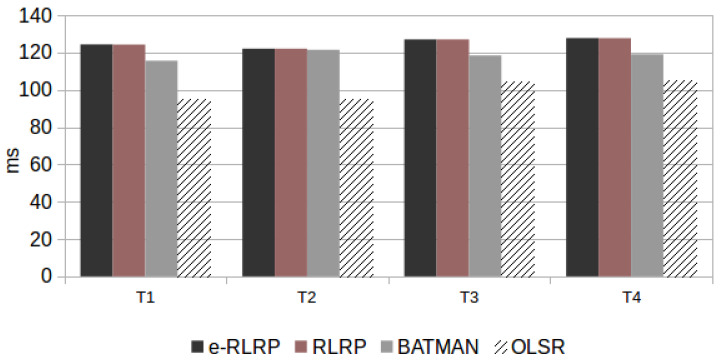
RTT Obtained in Scenario P (Programmed).

**Figure 11:**
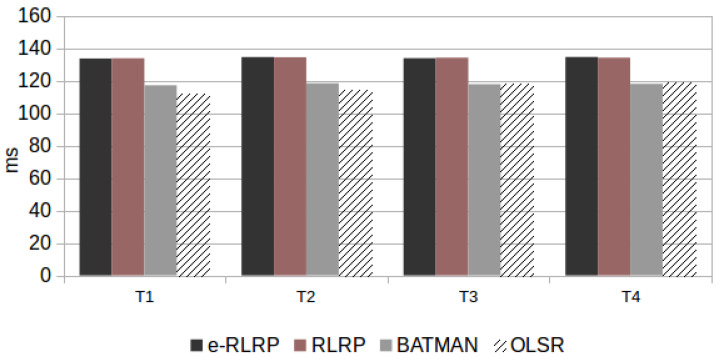
RTT Obtained in Scenario R (Random).

Additionally, the RTT parameter values obtained in scenario P is presented in Figure 10. Figure 11 shows the average of the RTT values of the scenarios R with also a single flow. These results represent the average values of two AMR-WB mode, because there was not difference between them.

Analyzing the results presented in Figure 10 and Figure 11, it is observed that the e-RLRP and RLRP presented the highest RTT values. This can be justified because they are implemented in user space on Linux using a dynamic Python interpreter. According to Reference [40], this implementation-type generates a great loss of performance mainly due to the high number of I/O operations that cause delays in the packet sending process. It is worth mentioning that this is a limitation generated by the language in which it was implemented and not by the code/project. Thus, the implementation of these both protocols had a restriction in this regard, that was reflected in RTT values obtained in the experimental tests. According to (6) and (7), delays in the network have a negative impact on speech quality predictions.

**Figure 12:**
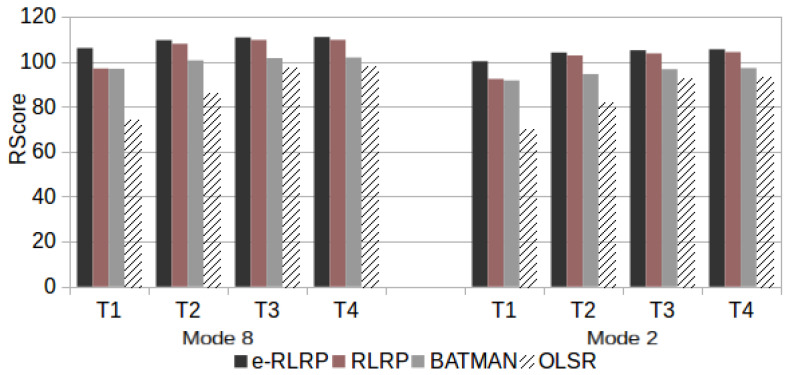
$R_{WB}$ Score in Scenario R with Three Drops.

**Figure 13:**
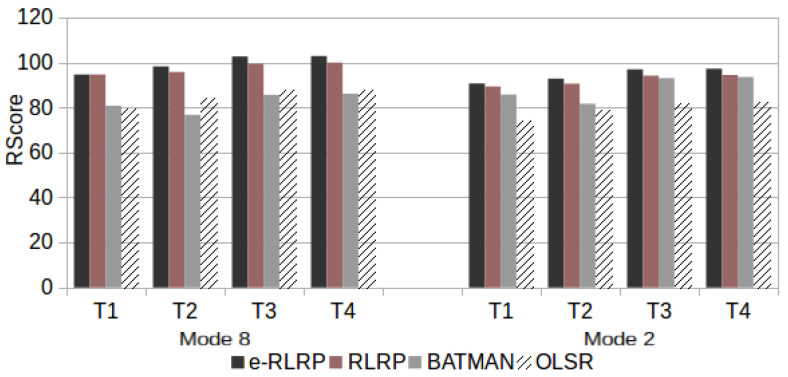
$R_{WB}$ Score in Scenario R with Five Drops.

**Figure 14:**
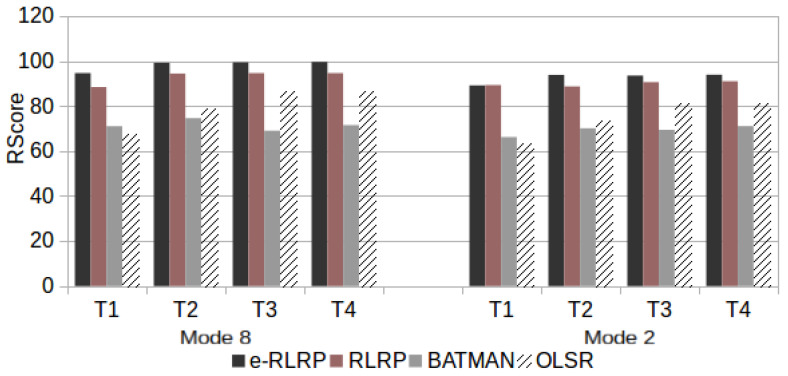
$R_{WB}$ Score in Scenario R with Seven Drops.

Finally, the speech communication quality was evaluated using (4)–(6) considering the Ppl and RTT values found in test scenarios that consider a single traffic flow with the topologies T1, T2, T3 and T4 used in this work. Figure 12 presents the $R_{WB}$ scores for scenario R with Three Drops, Figure 13 presents the $R_{WB}$ scores for Five Drops and Figure 14 with Seven Drops.

**Figure 15:**
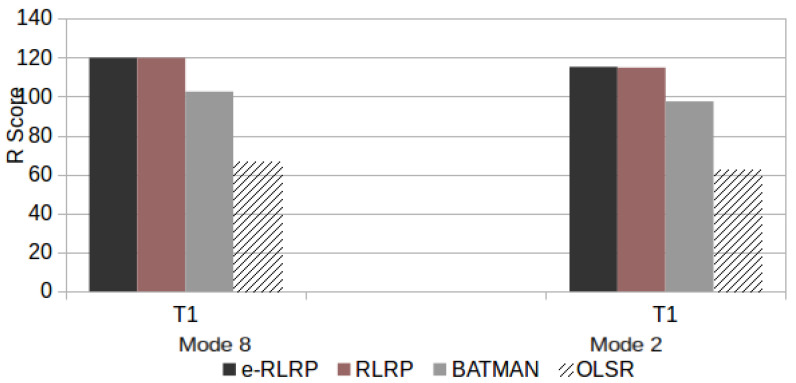
$R_{WB}$ Score in Scenario P.

Figure 15 presents the $R_{WB}$ scores for scenario P.

As can be observed from Figure 12, Figure 13, Figure 14 and Figure 15, the use of e-RLRP promotes a gain of $R_{WB}$ score in relation to those obtained by the RLRP, BATMAN and OLSR protocols. In some cases the gain in relation to OLSR is more to 90%. In relation to BATMAN, in some cases the gain is approximately 33%. In relation to the RLRP, the gain approaches 8%.

Therefore, RL in routing protocols improves the user’s QoE in a speech communication service. The e-RLRP not only reduces overhead but also provides a positive impact in the quality of VoIP communication, mainly because the *Ppl* is decreased.

## 6. Conclusions

In this work, the experimental results demonstrate that a routing protocol based on RL overcomes traditional protocols, such as BATMAN and OLSR, specifically in *Ppl* and throughput parameters. These network performance results prove the relevance of the RL-based routing protocols to improve the computer, and ad-hoc networks. However, the RL technique generates an extra overhead. Thus, the proposed and developed adjustment algorithm was able to reduce the network overhead in terms of reducing the number of control messages. The dynamic adjustment in the frequency of sending Hello messages provided a reduction of up to 18% overhead. This gain increases the network’s payload providing better network performance. The global performance of the proposed method was optimized using different configurations and parameter values, leading to a final configuration which was defined experimentally. In terms of throughput and *Ppl*, in most of the test scenarios used in this work the e-RLRP achieved better performance, specially with respect to the *Ppl* parameter. Therefore, it is demonstrated that the proposed solution reduces overhead and also improves the network conditions.

Reducing network overhead in conventional protocols is an important approach because it provides performance improvements. This approach is even more relevant when it is used by new routing techniques, such RL, that aim to improve network performance but it generates extra overhead. Thus, an important contribution of this work is to demonstrate that extra overhead can be reduced using the proposed dynamic adjustment function.

It is worth noting that in our experimental tests different network topologies and configurations were used, including different numbers of nodes and their drops, and also different numbers of traffic flows.

Also, experimental results show the impact of network performance parameters on the user’s QoE in the VoIP communication services. The e-RLRP obtained better values of $R_{WB}$ due to having lower Ppl values despite to have higher RTT values, which are calculated according to (6) and (7) defined in the *WB* E-model algorithm. In this case, it is observed that *Ppl* has a greater negative impact on speech quality than RTT, for the values obtained in the simulation scenarios considered in this research. Results indicate a quality improvement of more than 90% if compared to OLSR, and up to 8% if compared to RLRP. Therefore, it can be concluded that the RL-based routing protocols has a significant positive impact on user’s QoE in real-time communication services.

As a general conclusion, this research highlights the usefulness of incorporating machine-learning algorithms in routing protocols, specially for ad-hoc networks that recurrently present node drops. RL-based routing protocols can help to improve network conditions, and as a consequence, different communication applications are improved. In this work, only the VoIP service is evaluated, but in future works, video communication service will also be evaluated. Also, the implemented dynamic adjustment mechanism in the sending of Hello messages provided a performance improvement on the network, mainly by reducing overhead, which is an important approach to be applied in RL-based routing protocols.

In a future work, the proposed e-RLRP will be implemented in a real network environment to validate the performance results and potential benefits found in our simulation tests. Also, the inclusion of a scheduler or decentralized schedulers will be considered to work in conjunction with the e-RLRP algorithm in a future research, in which more complex and dynamic networks will be also implemented.

## Figures and Tables

**Table 1:** Relationship Between Reinforcement Learning (RL) and Conventional Routing Protocols.

RL Task	Routing Task
Agent	Source node
Set of actions	Neighbors set
Set of estimation values (Q)	Routing table
Agent Action	Send a packet to neighbors
Agent receives a reward	Node receives an ACK message

**Table 2:** Adaptive Multi-Rate Wideband (AMR-WB) Operation Modes and their Bit-rates, $I_{e}$ and Bpl Values.

AMR-WB Operation Modes	Number of Bits	Bit-Rate (kbps)	$I_{e}$	Bpl
0	132	6.60	41	ND
1	177	8.85	26	ND
2	253	12.65	13	4.3
3	285	14.25	10	ND
4	317	15.85	7	ND
5	365	18.25	5	ND
6	397	19.85	3	ND
7	461	23.05	1	ND
8	477	23.85	8	4.9

**Table 3:** Reward Header Format.

Field Name	Size (Bits)	Description
TYPE	4	Type ID of the header
ID	20	ID of the service message
NEG_REWARD_FLAG	1	Check reward
REWARD_VALUE	7	Value of the reward
MSG_HASH	32	ID of the data packet

**Table 4:** Hello Header Format.

Field Name	Size (Bits)	Description
TYPE	4	Type ID of the header
IPV4_COUNT	1	Number of IPv4 addresses
IPV6_COUNT	2	Number of IPv6 addresses
TX_COUNT	24	Number of frame re-broadcasts
GW_MODE	1	Indicates GateWay Mode
IPV4_ADDRESS	32	IPv4 address (if exists)
IPV6_ADDRESS_1	128	IPv6 address 1 (if exists)
IPV6_ADDRESS_2	128	IPv6 address 2 (if exists)
IPV6_ADDRESS_3	128	IPv6 address 3 (if exists)

**Table 5:** Broadcast Interval values to obtain overhead.

Broadcast Interval Value (s)	Overhead (MB)	Overhead Increase (%)
1.5	1.45	2.12% gain in relation to 2.0 s
1.0	1.48	4.22% gain in relation to 2.0 s
0.5	1.60	12.67% gain in relation to 2.0 s

**Table 6:** Nodes Set to Disconnect.

	T1	T2	T3	T4
Scenario P	D	-	-	-
Scenario R	ADG	ADGJ	ADGJL	ADGJNO

**Table 7:** Bit-rate After Adding RTP, UDP and IP headers.

AMR-WB Bit-Rate (kpbs)	Bit-Rate Considering RTP/UDP/IP Headers (kpbs)
12.65	28.65
23.85	39.85

**Table 8:** Overhead (kbps) obtained in Scenario without drops considering AMR-WB Modes 8 and 2.

Routing Protocol	Mode 8 (kbps)	Mode 2 (kbps)
e-RLRP	**2.29**	**2.24**
RLRP	2.71	2.65
Batman	6.60	6.30
OLSR	2.63	2.58

**Table 9:** Throughput (kbps) obtained in Scenario P considering AMR-WB Modes 2 and 8.

	AMR-WB Mode-8	AMR-WB Mode-2
	T1	T1
	(kbps)	(kbps)
e-RLRP	**39.80**	**28.60**
RLRP	39.79	28.60
Batman	39.28	28.25
OLSR	36.59	26.31

**Table 10:** *Ppl* (%) obtained in Scenario P considering AMR-WB Modes 2 and 8.

	AMR-WB Mode-8	AMR-WB Mode-2
	T1	T1
	(%)	(%)
e-RLRP	**0.04**	**0.03**
RLRP	0.05	0.05
Batman	1.3	1.23
OLSR	8.08	8.02

**Table 11:** Overhead (kbps) Obtained in Scenario P considering AMR-WB Modes 2 and 8.

	Mode 8 (kbps)	Mode 2 (kbps)
e-RLRP	**2.80**	**2.75**
RLRP	2.90	2.84
Batman	6.90	6.50
OLSR	2.88	2.80

**Table 12:** Throughput (kbps) Obtained in Scenario R with Three Drops considering AMR-WB Modes 2 and 8.

	AMR-WB Mode-8				AMR-WB Mode-2			
	T1	T2	T3	T4	T1	T2	T3	T4
	(kbps)	(kbps)	(kbps)	(kbps)	(kbps)	(kbps)	(kbps)	(kbps)
e-RLRP	**39.40**	**39.50**	**39.54**	**39.56**	**28.31**	**28.39**	**28.41**	**28.41**
RLRP	39.06	39.46	39.51	39.51	28.11	28.37	28.42	28.38
Batman	39.05	39.20	39.24	39.30	28.06	28.17	28.20	28.22
OLSR	37.57	38.5	39.09	39.14	27.06	27.74	28.11	28.12

**Table 13:** *Ppl* (%) Obtained in Scenario R with Three Drops considering AMR-WB Modes 2 and 8.

	AMR-WB Mode-8				AMR-WB Mode-2			
	T1	T2	T3	T4	T1	T2	T3	T4
	(%)	(%)	(%)	(%)	(%)	(%)	(%)	(%)
e-RLRP	**1.00**	**0.73**	**0.64**	**0.58**	**1.01**	**0.72**	**0.65**	**0.63**
RLRP	1.85	0.85	0.72	0.71	1.72	0.81	0.75	0.76
Batman	1.86	1.49	1.39	1.25	1.79	1.51	1.35	1.32
OLSR	5.60	3.25	1.78	1.65	5.40	3.02	1.71	1.67

**Table 14:** Overhead (kbps) Obtained in Scenario R with Three Drops considering AMR-WB Modes 2 and 8.

	AMR-WB Mode-8				AMR-WB Mode-2			
	T1	T2	T3	T4	T1	T2	T3	T4
	(kbps)	(kbps)	(kbps)	(kbps)	(kbps)	(kbps)	(kbps)	(kbps)
e-RLRP	**2.71**	**2.83**	**2.95**	**2.99**	**2.68**	**2.77**	**2.89**	**2.92**
RLRP	2.79	2.93	3.45	3.89	2.71	2.95	3.03	3.77
Batman	6.20	7.80	8.03	8.45	6.05	7.70	7.97	8.23
OLSR	2.74	2.94	2.99	3.27	2.70	2.86	2.95	3.18

**Table 15:** Throughpu (kbps) Obtained in Scenario R with Five Drops considering AMR-WB Modes 2 and 8.

	AMR-WB Mode-8				AMR-WB Mode-2			
	T1	T2	T3	T4	T1	T2	T3	T4
	(kbps)	(kbps)	(kbps)	(kbps)	(kbps)	(kbps)	(kbps)	(kbps)
e-RLRP	**38.98**	**39.11**	**39.28**	39.28	**28.06**	**28.20**	**28.23**	**28.21**
RLRP	38.97	39.01	39.16	39.23	28.01	28.05	28.16	28.19
Batman	38.48	38.20	38.88	38.95	27.68	27.47	27.87	27.99
OLSR	38.03	38.38	38.60	38.64	27.32	27.59	27.73	27.74

**Table 16:** *Ppl* (%) Obtained in Scenario R with Five Drops considering AMR-WB Modes 2 and 8.

	AMR-WB Mode-8				AMR-WB Mode-2			
	T1	T2	T3	T4	T1	T2	T3	T4
	(%)	(%)	(%)	(%)	(%)	(%)	(%)	(%)
e-RLRP	**2.10**	**1.71**	**1.29**	**1.30**	**1.90**	**1.67**	**1.28**	**1.25**
RLRP	**2.10**	1.98	1.60	1.54	2.05	1.91	1.54	1.50
Batman	3.30	4.01	2.30	2.10	3.20	3.91	2.50	2.30
OLSR	4.44	3.56	3.01	2.95	4.47	3.52	3.04	2.98

**Table 17:** Overhead (kbps) Obtained in Scenario R with Five Drops considering AMR-WB Modes 2 and 8.

	AMR-WB Mode-8				AMR-WB Mode-2			
	T1	T2	T3	T4	T1	T2	T3	T4
	(kbps)	(kbps)	(kbps)	(kbps)	(kbps)	(kbps)	(kbps)	(kbps)
e-RLRP	**2.90**	**2.98**	**3.01**	**3.03**	**2.86**	**2.94**	**2.98**	**3.02**
RLRP	2.94	3.14	3.88	3.95	2.92	3.09	3.78	3.90
Batman	11.05	11.20	11.40	11.48	11.01	11.00	11.30	11.38
OLSR	3.16	3.70	4.67	4.72	3.12	3.75	4.52	4.61

**Table 18:** Throughput (kbps) Obtained in Scenario R with Seven Drops considering AMR-WB Modes 2 and 8.

	AMR-WB Mode-8				AMR-WB Mode-2			
	T1	T2	T3	T4	T1	T2	T3	T4
	(kbps)	(kbps)	(kbps)	(kbps)	(kbps)	(kbps)	(kbps)	(kbps)
e-RLRP	**38.98**	**39.15**	**39.13**	39.19	**28.01**	**28.15**	**28.14**	**28.16**
RLRP	38.97	38.95	38.98	**39.01**	28.00	27.99	28.06	28.19
Batman	37.19	37.63	37.47	37.49	26.73	27.05	27.00	27.03
OLSR	36.77	37.98	38.52	38.55	26.40	27.30	27.71	27.73

**Table 19:** *Ppl* (%) Obtained in Scenario R with Seven Drops considering AMR-WB Modes 2 and 8.

	AMR-WB Mode-8				AMR-WB Mode-2			
	T1	T2	T3	T4	T1	T2	T3	T4
	(%)	(%)	(%)	(%)	(%)	(%)	(%)	(%)
e-RLRP	2.10	**1.61**	**1.59**	**1.45**	**2.01**	**1.57**	**1.60**	**1.54**
RLRP	**2.09**	2.13	2.10	1.55	2.05	2.12	1.90	1.83
Batman	6.55	5.45	5.85	5.62	6.56	5.39	5.60	5.49
OLSR	7.60	4.56	3.20	3.10	7.70	4.54	3.10	3.01

**Table 20:** Overhead (kbps) Obtained in Scenario R with Seven Drops considering AMR-WB Modes 2 and 8.

	AMR-WB Mode-8				AMR-WB Mode-2			
	T1	T2	T3	T4	T1	T2	T3	T4
	(kbps)	(kbps)	(kbps)	(kbps)	(kbps)	(kbps)	(kbps)	(kbps)
e-RLRP	**2.98**	**3.41**	**4.84**	**4.99**	**2.94**	**3.25**	**4.10**	**4.96**
RLRP	3.10	3.64	5.29	5.45	3.01	3.39	5.36	5.25
Batman	11.12	11.15	12.10	12.35	11.21	11.13	12.01	12.49
OLSR	3.26	3.79	5.10	5.17	3.20	3.85	4.99	5.01

**Table 21:** Throughput (kbps) Obtained in Scenario R with Three Flows considering AMR-WB Mode 8.

	Mode 8					
	T3			T4		
	3 Drops	5 Drops	7 Drops	3 Drops	5 Drops	7 Drops
	CE/FB/IH	CE/FB/IH	CE/FB/IH	CE/FB/IH	CE/FB/IH	CE/FB/IH
	(kbps)	(kbps)	(kbps)	(kbps)	(kbps)	(kbps)
e-RLRP	**38.50**/**38.38**/38.20	**38.16**/38.11/**38.33**	**37.81**/**37.60**/**38.10**	**38.49**/**38.40**/**38.27**	**38.15**/**38.17**/**38.32**	**37.91**/**37.77**/**38.06**
RLRP	38.33/38.19/38.18	38.10/**38.14**/38.28	37.77/37.59/37.50	38.40/38.32/38.25	38.14/38.16/38.29	37.87/37.75/37.83
Batman	38.17/38.18/**38.37**	37.60/37.71/37.72	36.89/36.90/37.77	38.18/38.22/38.26	37.71/37.83/37.79	36.83/37.02/36.95
OLSR	37.93/38.11/38.18	37.32/37.84/38.01	37.46/37.59/37.44	38.04/38.22/38.26	37.48/37.96/38.06	37.52/37.66/37.56

**Table 22:** *Ppl* (%) Obtained in Scenario R with Three Flows considering AMR-WB Mode 8.

	Mode 8					
	T3			T4		
	3 Drops	5 Drops	7 Drops	3 Drops	5 Drops	7 Drops
	CE/FB/IH	CE/FB/IH	CE/FB/IH	CE/FB/IH	CE/FB/IH	CE/FB/IH
	(%)	(%)	(%)	(%)	(%)	(%)
e-RLRP	**0.69**/**0.63**/1.03	1.40/**1.10**/**0.82**	**2.05**/**2.63**/**1.99**	**0.51/0.51/0.85**	**1.17/1.10/0.72**	**1.80/2.20/1.40**
RLRP	0.71/1.06/1.09	**1.31**/1.19/0.85	2.15/2.66/2.86	0.52/0.74/0.91	1.20/1.15/0.80	1.90/**2.20**/2.00
Batman	1.41/1.18/**1.01**	2.70/2.40/2.28	5.40/4.60/5.10	1.10/0.98/0.88	2.30/2.00/2.10	4.60/4.10/4.20
OLSR	1.75/1.27/1.1	3.41/1.96/1.52	2.95/2.68/3.01	1.45/0.98/0.89	2.90/1.67/1.40	2.80/2.45/2.70

**Table 23:** Overhead (kbps) Obtained in Scenario R with Three flows considering AMR-WB Mode 8.

	Mode 8					
	T3			T4		
	3 Drops	5 Drops	7 Drops	3 Drops	5 Drops	7 Drops
	(kbps)	(kbps)	(kbps)	(kbps)	(kbps)	(kbps)
e-RLRP	**2.78**	**3.33**	**5.92**	**2.81**	**3.35**	**6.17**
RLRP	4.78	4.72	7.95	4.90	4.82	6.41
Batman	9.90	12.20	15.45	10.20	11.98	14.85
OLSR	3.92	5.10	6.80	4.10	5.33	6.21

**Table 24:** Throughput (kbps) Obtained in Scenario R with Three Flows considering AMR-WB Mode 2.

	Mode 2					
	T3			T4		
	3 Drops	5 Drops	7 Drops	3 Drops	5 Drops	7 Drops
	CE/FB/IH	CE/FB/IH	CE/FB/IH	CE/FB/IH	CE/FB/IH	CE/FB/IH
	(kbps)	(kbps)	(kbps)	(kbps)	(kbps)	(kbps)
e-RLRP	**27.84**/**27.87**/**27.77**	**27.86**/27.65/**27.82**	**27.48**/**27.31**/**27.67**	**27.85**/**27.86**/**27.76**	**27.65**/**27.71**/**27.84**	**27.50**/**27.35**/**27.69**
RLRP	27.83/27.71/27.73	27.80/**27.70**/27.66	27.46/27.30/27.23	27.84/ 27.76/27.75	27.65/27.74/27.82	27.48/27.34/27.20
Batman	27.69/27.74/27.76	27.60/27.63/27.66	26.69/26.97/26.87	27.70/27.75/27.75	27.63/27.68/27.71	26.87/26.91/26.80
OLSR	27.57/27.66/**27.77**	27.06/27.49/27.61	27.23/27.29/27.20	27.61/27.65/27.71	27.24/27.48/27.65	27.21/27.24/27.23

**Table 25:** *Ppl* (%) Obtained in Scenario R with Three Flows considering AMR-WB Mode 2.

	Mode 2					
	T3			T4		
	3 Drops	5 Drops	7 Drops	3 Drops	5 Drops	7 Drops
	CE/FB/IH	CE/FB/IH	CE/FB/IH	CE/FB/IH	CE/FB/IH	CE/FB/IH
	(%)	(%)	(%)	(%)	(%)	(%)
e-RLRP	**0.64**/**0.56**/**0.99**	**1.14**/1.27/**0.71**	**1.95**/**2.58**/**1.25**	**0.62**/**0.54**/**0.85**	**1.32**/**1.01**/**0.59**	**1.86**/**2.39**/**1.19**
RLRP	0.68/1.03/1.11	1.20/**1.15**/0.78	2.02/2.59/2.84	0.64/0.95/0.98	1.32/1.02/0.72	1.95/2.42/2.69
Batman	1.19/1.01/1.03	1.70/1.40/1.28	4.50/4.10/4.30	1.15/ 0.98/0.96	1.42/1.22/1.10	4.10/3.98/4.35
OLSR	1.62/1.28/0.90	3.45/1.90/1.47	2.83/2.60/2.94	1.49/1.23/0.86	2.67/1.90/1.32	2.71/2.62/2.83

**Table 26:** Overhead (kbps) Obtained in Scenario R with Three flows considering AMR-WB Mode 2.

	Mode 2					
	T3			T4		
	3 Drops	5 Drops	7 Drops	3 Drops	5 Drops	7 Drops
	(kbps)	(kbps)	(kbps)	(kbps)	(kbps)	(kbps)
e-RLRP	**2.62**	**3.09**	**6.89**	**3.02**	**2.97**	**6.14**
RLRP	4.16	4.76	6.92	4.21	4.80	6.62
Batman	9.70	11.45	14.26	9.23	11.52	13.52
OLSR	3.86	4.95	5.76	3.49	4.88	6.77

**Table 27:** Throughput (kbps) Obtained in Scenario R with Four Flows considering AMR-WB Mode 8.

	Mode 8					
	T3			T4		
	3 Drops	5 Drops	7 Drops	3 Drops	5 Drops	7 Drops
	CE/FB/IH	CE/FB/IH	CE/FB/IH	CE/FB/IH	CE/FB/IH	CE/FB/IH
	(kbps)	(kbps)	(kbps)	(kbps)	(kbps)	(kbps)
e-RLRP	**38.40**/**38.23**/**38.29**/39.8	**38.23**/37.98/**38.26**/**39.8**	**38.40**/**37.93**/**38.26**/39.8	**38.41**/**38.29**/**38.29**/39.8	**38.28**/**38.09**/**38.21**/39.8	**38.04**/**37.87**/**37.86**/39.8
RLRP	38.37/**38.23**/38.27/39.01	38.14/**38.16**/38.20/39.6	37.89/37.79/38.18/39.8	38.39/38.28/38.29/39.8	38.15/38.07/38.18/39.8	38.02/37.82/37.81/39.8
Batman	37.99/38.06/38.15/39.7	37.60/37.49/37.53/39.8	36.52/36.71/36.79/39.8	38.04/38.10/38.22/39.8	37.71/37.54/37.51/39.8	36.72/36.92/36.81/39.8
OLSR	38.22/38.18/38.21/39.8	37.40/37.61/38.07/39.8	37.45/37.71/37.71/39.8	38.25/38.22/38.25/39.8	37.46/37.59/38.10/39.8	37.46/37.60/37.62/39.8

**Table 28:** *Ppl* (%) Obtained in Scenario R with Four Flows considering AMR-WB Mode 8.

	Mode 8					
	T3			T4		
	3 Drops	5 Drops	7 Drops	3 Drops	5 Drops	7 Drops
	CE/FB/IH	CE/FB/IH	CE/FB/IH	CE/FB/IH	CE/FB/IH	CE/FB/IH
	(%)	(%)	(%)	(%)	(%)	(%)
e-RLRP	**0.52**/**0.88**/**0.92**/0	**0.96**/**1.10**/**1.14**/0	**1.79**/**2.30**/**1.85**/0	**0.49**/**0.75**/ **0.81**/0	**0.83**/**1.30**/**1.01**/0	**1.45**/**1.89**/**1.81**/0
RLRP	0.61/0.95/0.96/0	1.22/1.25/1.15/0	1.83/2,31/1.93/0	0.55/0.84/**0.81**/0	1.17/1.35/1.10/0	1.50/1.96/1.89/0
Batman	1.59/1.41/1.16/0	2.58/2.89/2.78/0	5.40/4.90/4.70/0	1.46/ 1.31/0.98/0	2.30/2.76/2.65/0	4.78/4.30/4.65/0
OLSR	0.98/1.10/1.02/0	3.11/2.57/1.38/0	3.08/2.68/2.64/0	0.92/0.98/0.91/0	2.95/2.63/1.29/0	2.95/2.59/2.43/0

**Table 29:** Overhead (kbps) Obtained in Scenario R with Four flows considering AMR-WB Mode 8.

	Mode 8					
	T3			T4		
	3 Drops	5 Drops	7 Drops	3 Drops	5 Drops	7 Drops
	(kbps)	(kbps)	(kbps)	(kbps)	(kbps)	(kbps)
e-RLRP	**3.15**	**4.19**	**4.20**	**3.25**	**4.37**	**4.60**
RLRP	3.45	5.45	4.92	3.49	5.6	5.10
Batman	9.22	10.20	13.03	9.3	10.10	13.02
OLSR	3.22	4.72	5.30	3.33	4.68	5.90

**Table 30:** Throughput (kbps) Obtained in Scenario R with Four Flows considering AMR-WB Mode 2.

	Mode 2					
	T3			T4		
	3 Drops	5 Drops	7 Drops	3 Drops	5 Drops	7 Drops
	CE/FB/IH	CE/FB/IH	CE/FB/IH	CE/FB/IH	CE/FB/IH	CE/FB/IH
	(kbps)	(kbps)	(kbps)	(kbps)	(kbps)	(kbps)
e-RLRP	**27.58**/**27.48**/**27.50**/28.6	**27.51**/**27.59**/**27.43**/28.6	**27.29**/**27.15**/**27.48**/28.6	**27.88**/**27.82**/**27.79**/28.6	**27.81**/**27.70**/**27.74**/28.6	**27.71**/**27.41**/**27.65**/28.6
RLRP	27.57/**27.48**/27.49/28.6	27.35/27.42/27.41/28.6	27.20/27.13/27.40/28.6	27.85/27.77/27.78/28.6	27.80/27.62/27.72/28.6	27.68/27.37/27.62/28.5
Batman	27.32/27.31/27.39/28.6	26.99/26.94/26.91/28.5	26.27/26.52/26.60/28.6	27.61/27.64/27.73/28.6	27.35/27.31/27.33/28.6	26.62/26.87/26.93/28.6
OLSR	27.29/27.38/27.49/28.6	26.78/27.21/27.33/28.6	26.95/27.02/26.92/28.5	27.61/27.64/27.73/28.6	27.35/27.31/27.33/28.6	26.62/26.87/26.93/28.6

**Table 31:** *Ppl* (%) Obtained in Scenario R with Four Flows considering AMR-WB Mode 2.

	Mode 2					
	T3			T4		
	3 Drops	5 Drops	7 Drops	3 Drops	5 Drops	7 Drops
	CE/FB/IH	CE/FB/IH	CE/FB/IH	CE/FB/IH	CE/FB/IH	CE/FB/IH
	(%)	(%)	(%)	(%)	(%)	(%)
e-RLRP	**0.58**/**0.90**/**0.85**/0	**0.87**/**1.06**/**1.11**/0	**1.62**/**2.10**/**1.55**/0	**0.52**/**0.76**/**0.83**/0	**0.75**/**1.40**/**1.01**/0	**1.17**/**2.20**/**1.32**/0
RLRP	0.59/0.91/0.89/0	1.38/1.26/1.19/0	1.93/2,20/1.63/0	0.62/0.84/ 0.84/0	**0.75**/1.41/1.05/0	1.23/2.32/1.43/0
Batman	1.52/1.55/1.26/0	2.70/2.86/2.98/0	5.30/4.40/4.10/0	1.49/1.35/1.03/0	2.41/2.56/2.49/0	5.01/4.1/3.89/0
OLSR	1.62/1.28/0.90/0	3.45/1.90/1.47/0	2.83/2.60/2.94/0	1.49/1.35/1.03/0	2.41/2.56/2.49/0	5.01/4.1/3.89/0

**Table 32:** Overhead (kbps) Obtained in Scenario R with Four flows considering AMR-WB Mode 2.

	Mode 2					
	T3			T4		
	3 Drops	5 Drops	7 Drops	3 Drops	5 Drops	7 Drops
	(kbps)	(kbps)	(kbps)	(kbps)	(kbps)	(kbps)
e-RLRP	**2.96**	**4.26**	**4.13**	**3.28**	**4.10**	**4.46**
RLRP	3.88	5.25	4.61	3.72	4.55	4.88
Batman	8.26	9.04	11.65	8.25	9.16	11.33
OLSR	3.06	4.66	5.30	3.95	4.68	5.11
